# The Membrane Steps of Bacterial Cell Wall Synthesis as Antibiotic Targets

**DOI:** 10.3390/antibiotics5030028

**Published:** 2016-08-26

**Authors:** Yao Liu, Eefjan Breukink

**Affiliations:** Department of Membrane Biochemistry and Biophysics, Utrecht University, Utrecht 3584 CH, The Netherlands; y.liu3@uu.nl

**Keywords:** peptidoglycan, MraY, MurG, inhibition, antibiotics, mechanism, target

## Abstract

Peptidoglycan is the major component of the cell envelope of virtually all bacteria. It has structural roles and acts as a selective sieve for molecules from the outer environment. Peptidoglycan synthesis is therefore one of the most important biogenesis pathways in bacteria and has been studied extensively over the last twenty years. The pathway starts in the cytoplasm, continues in the cytoplasmic membrane and finishes in the periplasmic space, where the precursor is polymerized into the peptidoglycan layer. A number of proteins involved in this pathway, such as the Mur enzymes and the penicillin binding proteins (PBPs), have been studied and regarded as good targets for antibiotics. The present review focuses on the membrane steps of peptidoglycan synthesis that involve two enzymes, MraY and MurG, the inhibitors of these enzymes and the inhibition mechanisms. We also discuss the challenges of targeting these two cytoplasmic membrane (associated) proteins in bacterial cells and the perspectives on how to overcome the issues.

## 1. Introduction

Bacteria have evolved to survive a considerable variety of environments and can develop resistance to various antibacterial reagents rapidly. This ability of bacteria presents a big challenge in treating infections, especially in hospitals. The peptidoglycan layer that is in almost all bacteria lends structural strength and provides a protective barrier for the bacterium [1]. The peptidoglycan layer is composed of polysaccharides with alternating *N*-acetylglucosamine (GlcNAc) and *N*-acetylmuramic acid (MurNAc) saccharide groups. Between three and five amino acids, typically a pentapeptide with a sequence of l-Ala-γ-d-Glu-l-lysine (or -*meso*-diaminopimelic acid)-d-Ala-d-Ala [2], are attached to the MurNAc group. These peptide chains can be crosslinked with each other.

Peptidoglycan synthesis occurs in three distinctive compartments of bacteria, namely the cytoplasm, the cytoplasmic membrane and the periplasmic space [3]. It starts in the cytoplasm, where the nucleotide precursors are synthesized, i.e., UDP-GlcNAc is synthesized from fructose-6-phosphate by the Glm enzymes [4], and UDP-*N*-acetylmuramyl-pentapeptide (UDP-Mpp) is synthesized by the Mur enzymes (MurA, MurB, MurC, MurD, MurE and MurF) [5] from UDP-GlcNAc. The synthesis of the membrane-embedded undecaprenyl phosphate takes place on the cytoplasmic side. This is performed by undecaprenyl pyrophosphate synthase (UppS) catalyzing the consecutive condensation reactions of a farnesyl pyrophosphate (FPP) with eight isopentenyl pyrophosphates (IPP), in which new *cis*-double bonds are formed [6]. The resulting undecaprenyl pyrophosphate (C55-PP) is dephosphorylated by undecaprenyl pyrophosphate phosphatase (UppP) to produce undecaprenyl phosphate (C55-P) [7]. UDP-Mpp and C55-P are the two substrates of the integral membrane enzyme phospho-MurNAc-pentapeptide translocase (MraY). MraY catalyzes the first membrane step of peptidoglycan synthesis by transferring the phospho-MurNAc-pentapeptide moiety from UDP-Mpp to C55-P and yields uridine-monophosphate (UMP) and undecaprenyl-pyrophosphoryl-MurNAc-pentapeptide, typically referred to as Lipid I [8]. Although the use of C55-P as the lipid carrier is the common rule in bacteria, there is evidence that the shorter chain decaprenyl phosphate and nonaprenyl phosphate homologues assist glycan translocation in some species [9,10]. Moreover, C55-P is also shared by other cell wall pathways, such as the wall teichoic acid synthesis and capsular polysaccharide synthesis in *Staphylococcus aureus* [11,12,13,14]. Since C55-P exists in bacterial cells in very limited amounts, the synthesis of these different components is highly integrated and coordinated temporally.

After Lipid I synthesis, the glycosyltransferase MurG transfers a GlcNAc moiety from UDP-GlcNAc to Lipid I to produce undecaprenyl-pyrophosphoryl-MurNAc-(pentapeptide)- GlcNAc. This is usually referred to as Lipid II, which is subsequently transported by a flippase from the inner side of the membrane to the outer side [15,16,17,18] where polymerization to give a peptidoglycan layer takes place. The proteins that catalyze the last steps of the formation of the peptidoglycan layer have been researched in detail and include the bifunctional penicillin binding proteins (PBPs), e.g., PBP1A and PBP1B of Gram-negative *Escherichia coli* [19] and PBP4 of Gram-positive *Listeria monocytogenes* [20]. The transglycosylase domain of the PBPs polymerizes the sugar moieties of Lipid II to produce glycan strands, where the transpeptidase domain links the peptides to form a 3D network. These actions ultimately result in the peptidoglycan layer that is responsible for the shape and rigidity of the bacterial cell [21].

Destruction of the peptidoglycan layer brings about loss of integrity and can lead to cell death by bursting. Some most successful and widely-used antibiotics, such as the β-lactams and glycopeptide antibiotics [22,23,24,25,26], have targets in the peptidoglycan synthesis pathway. Although studied in some detail, this pathway can still be exploited for novel antibacterial compounds. Research into the peptidoglycan biogenesis can therefore form part of our response to the ever-present problem of resistance to antibiotics, as well as improve our understanding of bacterial physiology.

Several reviews have been published dealing with different stages of peptidoglycan biogenesis; the present review focuses on the membrane steps of this pathway, summarizing the recent advances in the research of structure, function, inhibition mechanisms and the attempts to develop inhibitors of the essential enzymes, MraY and MurG. There are several enzymes, including WecA, TagO and WbcO, of the PNPT family that fulfill similar roles as MraY or MurG, in some bacterial species. These will not be discussed in the present review to focus on the more commonplace types of bacterium and to keep our messages concise. Extensive reviews dealing with similar topics, such as MraY inhibitors and peptidoglycan lipid intermediates, have been published by different groups in 2006 [27] and 2007 [2]; the present review therefore focuses on the advances that were made thereafter with a brief introduction and overview of the earlier knowledge.

## 2. MraY

**Figure 1:**
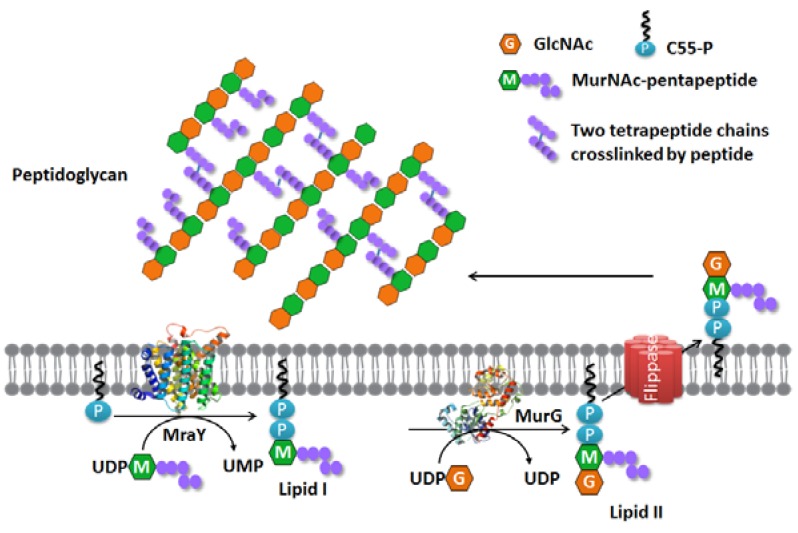
Membrane steps of the bacterial peptidoglycan synthesis pathway.

The first evidence of phospho-MurNAc-pentapeptide translocase (MraY) and its function was collected in 1965 when an active membrane fraction was prepared in vitro that could successfully produce Lipid I (Figure 1) [28]. The enzyme that was responsible for the production of Lipid I was often referred to as translocase I until 1991, when the *MraY* (mra: murein synthesis gene cluster a) gene for the enzyme responsible was identified in *E. coli* [29].

### 2.1. Biochemical Characterization of MraY

In-depth biochemical analysis of protein function relies on the overexpression and purification of the desired protein and in a manner that retains its enzymatic activity. Early studies on MraY relied on crude membrane preparations of MraY, because production, as well as detailed investigations of the biochemical properties of MraY had been long held back by the hydrophobic nature of this enzyme. For example, a method of overexpressing *E. coli* MraY and solubilizing the membranes using Triton X-100 was reported [30,31]. It was demonstrated that the protein was produced at a concentration of 4 mg/mL, but it did not undergo a purification step. Using these protein-enriched membrane preparations, the binding modes of several MraY inhibitors, such as mureidomycin A, tunicamycin and liposidomycin B (see Section 2.3), were studied [30]. It was found that the nucleoside antibiotics displayed different modes of action, being competitive either with the nucleotide substrate UDP-Mpp or the lipid substrate C55-P. The information was yet limited on the binding mode since no structural model of the protein was available.

The purification of the Gram-positive *Bacillus subtilis* MraY (BsMraY) to homogeneity in the milligram range (from a 5-L cell culture) was first achieved by using an *n*-dodecyl-β-d-maltoside (DDM) detergent system [32]. Bouhss et al. [32] tested different detergent systems for their influence on the MraY activity; the ionic detergent with a low aggregation number, i.e., *N*-lauroyl sarcosine, was identified as the best detergent for a Lipid I synthesis assay using radiolabeled UDP-MurNAc-[^14^C]pentapeptide. The development of the purification method for BsMraY allowed the study of its kinetics. The K_M_ value of BsMraY was obtained by varying the concentration of either substrate while keeping the other at a fixed value. It was reported that the K_M_ values of pure BsMraY for its substrates UDP-Mpp and C55-P were 1.0 ± 0.3 mM and 0.16 ± 0.08 mM, respectively [32]. The catalytic constant K_cat_ was 320 ± 34 min^−1^. This result was later challenged by a report of a K_M_ value of pure BsMraY for UDP-Mpp being 36.2 ± 3.6 µM [33]. However, the two studies used different concentrations of the lipid carrier C55-P in the synthesis reaction, namely 1.1 mM in [32] and 50 µM in [33], suggesting that the true K_M_ value of BsMraY for UDP-Mpp is different from these apparent K_M_ values. Indeed, more recent kinetics studies on BsMraY showed that the apparent affinity of either substrate to the enzyme is dependent on the concentration of the other [34,35]. This characteristic has been overlooked by the other published studies.

Further investigations on the enzymatic mechanism of BsMraY were carried out using single mutants of invariant or highly-conserved polar residues (all in the cytoplasmic loops) using site-directed mutagenesis followed by detailed analysis of the mutant proteins [36]. The importance of highly-conserved aspartate residues, namely D98 and D99 in BsMraY (corresponding to D115 and D116 in *E. coli* MraY (EcMraY)), were addressed in this study [36]. It was the first time that MraY catalysis was studied with purified enzyme, both the wild-type and its mutants, without the interference of other contaminant enzymes or traces of C55-P in a membrane preparation. Fourteen single mutations that led to significant loss of activity revealed the involvement of these residues in the catalytic role of MraY. However, it was also observed that these mutants did not impair the binding of MraY to its nucleotide substrate, UDP-Mpp. Al-Dabbagh et al. [36] also proposed the involvement of D98 in deprotonation of the lipid substrate, C55-P, due to the clear influence of pH on the D98N mutant, as this mutant showed maximum activity at pH 9.0–9.4, where the wild-type worked optimally at pH 7.6. This suggests that the translocase reaction proceeds through a nucleophilic attack of phosphate oxyanion from the deprotonated C55-P onto the β-phosphate via the acidic residue (D98). Based on these findings, the authors support the proposed one-step catalytic mechanism for MraY. It is worth noting that in this study, the kinetic values of BsMraY and its mutants were obtained by varying UDP-Mpp concentration between 3 and 6 mM while keeping C55-P at 1.1 mM. These concentrations are very high, and the K_M_ values obtained were much higher than those of our own kinetic study [34]. The K_cat_ value of the H289R mutant reported in this study decreased by three orders of magnitude. However, in our own studies, the H289R mutant is virtually inactive, and it was impossible to determine the kinetic values.

The success of isolating and purifying BsMraY from Gram-positive species was not matched in studies of Gram-negative ones. A cell-free overexpression system coupled with detergent solubilization increased the yield of EcMraY considerably, giving yields in the milligram range from just a few milliliters of the cell-free reaction volumes [33,37,38]. However, EcMraY was found to form aggregates in the presence of a range of detergents, and any purified material was inactive [38]. The cell-free expression system was useful for investigating the role of lipid composition on EcMraY function. It was discovered that the activity of EcMraY is highly dependent on the presence of particular phospholipids [33]. Anionic and glycerol-containing lipid head groups, such as phosphatidylglycerol and cardiolipin, were shown to be essential for the functionality of EcMraY [39]. This was consistent with the research from the 1970s that showed MraY from *Micrococcus luteus* that was solubilized with Triton X-100 required neutral and polar lipids in order to remain active [40]. Notably, although a polar lipid was required for the synthesis of Lipid I, neither was required for the exchange reaction between UMP and UDP-Mpp [40]. The lack of structural information at the time limited the understanding of the mechanism of MraY, which made the reasons for the dependence on polar lipids unclear. Recent observations [34,35] indicate that an exchange reaction between UMP and UDP-Mpp does not occur unless C55-P is present, and the previously-suggested covalent enzyme-substrate intermediate, MraY-phospho-MurNAc-pp, is questionable [41]. Lloyd et al. [41] claimed that an intermediate was trapped in the reaction mixture without the addition of C55-P, an *E. coli* membrane preparation was used instead of pure MraY enzyme; therefore, the presence of a small amount of C55-P cannot be excluded.

### 2.2. Structural Characterization of MraY

**Figure 2:**
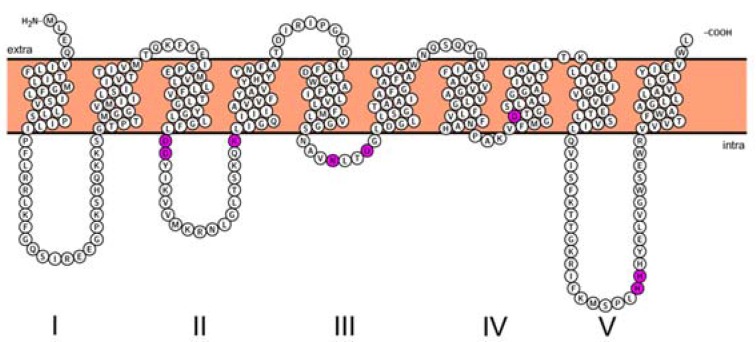
Topology maps of MraY from *B. subtilis*. Highly-conserved residues (D98, D99, K116, N171, D174, D231, H289 and H290) are highlighted in pink. The figure is rendered with the Protter web service [44]. Topology of other MraY species can be found in the Supplementary Information.

There was almost a 20-year gap between the discovery of the MraY enzyme (from *Staphylococcus aureus*) [42] in 1973 and the elucidation of the exact sequence of this protein (from *E. coli*) [29] in 1991. Alternating hydrophobic and hydrophilic fragments of amino acid sequences in MraY indicate that the enzyme spans the cytoplasmic membrane several times [29]. The topology maps of MraY from two different species, namely the Gram-positive *Staphylococcus aureus* and the Gram-negative *E. coli*, were later reported [43]. β-Lactamase fusions to different domains of MraY were constructed and expressed in *E. coli* DH5α cells. The resistance or sensitivity of the cells expressing different hybrids to ampicillin revealed the orientation of the different domains of MraY. It became clear that MraY has 10 transmembrane domains, and both its N- and C-termini are located in the periplasm across species (Figure 2). Since the precursors for Lipid I are synthesized in the cytoplasm, it follows that the active site of MraY is exposed to the cytoplasm, as well. It was proposed that cytosolic loops II, III and IV are responsible for the binding to UDP-Mpp, as they contained highly-conserved residues that are common for the polyprenyl-phosphate *N*-acetyl hexosamine 1-phosphate transferase (PNPT) super family, while loops I and V were likely involved in the interaction with other proteins of the peptidoglycan synthesis pathway given that the sequences were strictly specific to MraY orthologues.

**Figure 3:**
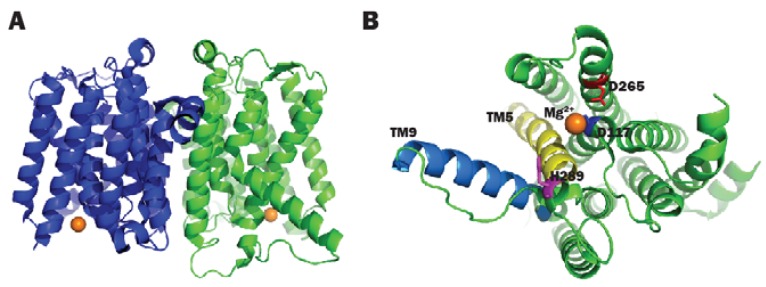
(**A**) 3D structure of AaMraY; (**B**) Close-up view of TM5 (helix shown in blue), TM9b (helix shown in cyan), the HHH motif (shown as rainbow sticks) in loop E, the Mg^2+^ (shown as an orange sphere) and the essential D265 of AaMraY. Images obtained and rendered with Pymol using 4J72.pdb.

**Figure 4:**
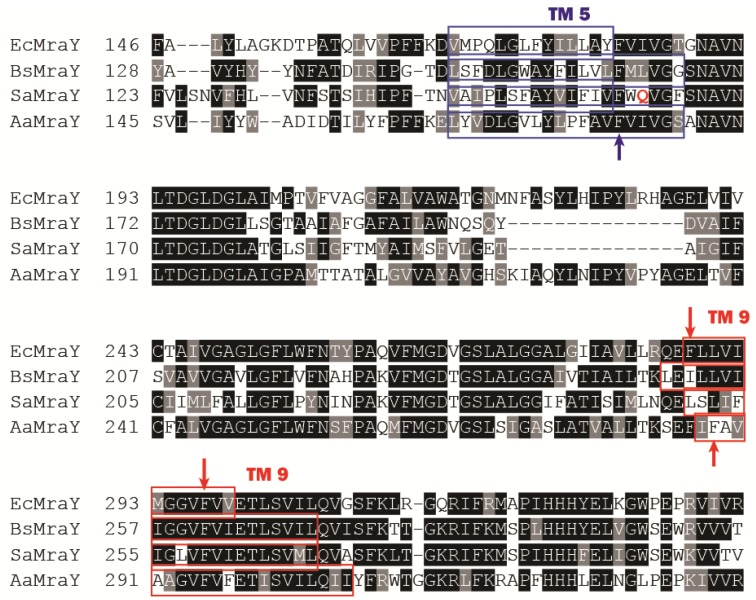
Partial sequence alignment of MraY from four different species (Ec = *E. coli*; Sa = *S. aureus*; Bs = *B. subtilis*; Aa = *A. aeolicus*). Some essential phenylalanine residues are indicated with an arrow. The polar Gln residue in *S. aureus* MraY is marked in red.

As briefly mentioned in the previous section, a number of conserved residues distributed all over the cytoplasmic loops of MraY were proven to be catalytically important taking BsMraY as a model (D98, D99, K116, N171, D174, D231, H289 and H290) (Figure 2) [36]. This suggests that all of the cytoplasmic loops are involved in maintaining the UDP-Mpp in an efficient configuration for catalysis. Two biochemistry-based catalytic models were proposed. One is a two-step model in which the MraY-p-MurNAc-pentapeptide intermediate is formed very rapidly to account for an exchange reaction [45,46]; the second is a one-step model that involves a direct attack of the phosphate oxyanion onto UDP-Mpp [36]. Unfortunately, as no 3D structure of any MraY isoform or even any member of the PNPT super family was available, it was not possible to be conclusive about by which mechanism this reaction proceeds. Many unanswered questions remained regarding how the other important residues affect the substrate binding and the catalytic process of MraY. Recently, Chung and his co-workers [8] reported the crystal structure of MraY from *Aquifex aeolicus* (AaMraY) at 3.3 Å resolution. The observation that the N and C termini are on the periplasmic side is consistent with previous studies of the topology of EcMraY, SaMraY and BsMraY. They also showed that AaMraY was crystallized as a dimer, and a large tunnel formed by the helices of the dimer interface could facilitate positioning of the lipid carrier C55-P. Moreover, the evidence for a cleft formed by the cytoplasmic and inner leaflet regions of transmembrane helices 3, 4, 5, 8 and 9b led to the suggestion that this was the active site of AaMraY. The cleft is deepened by the amphipathic helix TM9b protruding towards the cytoplasmic membrane, which is connected with a highly-conserved HHH motif (amino acid sequence: PXHHHXEXXG) in the fifth cytoplasmic loop, pointing towards the region where Mg^2+^ is bound (Figure 3). The activity of MraY is highly dependent on the presence of its co-factor Mg^2+^ [27,47]. Mg^2+^ binds close to D265 (Figure 3), which is a catalytic residue corresponding to D265 of BsMraY, D267 of EcMraY and D229 of SaMraY (Figure 4). It was suggested that the cleft is the essential binding site for the nucleotide substrate, UDP-Mpp. Mutagenesis of the residue showed the importance of D117 (corresponding to D98 in BsMraY and D115 in EcMraY) in binding to the lipid substrate, C55-P. Based on these findings, the study of Chung et al. supported the proposal that D117 deprotonates the phosphate moiety of C55-P allowing a direct nucleophile attack on UDP-Mpp. This evidence suggests that a one-step MraY catalysis mechanism is more likely than a two-step mechanism. Chung et al. [8] predicted that C55-P with a lipid chain longer than the thickness of the membrane needs to bend sharply to allow the phosphate moiety to reach D117, though there is no direct evidence as to how this is actually achieved. Furthermore, no clear catalytic role was attributed to the highly-conserved H324 (corresponding to H289 in BsMraY). It was proposed that 13 amino acids, including the HHH motif in the cytoplasmic loop, are unique to the MraY family and contribute to the substrate specificity towards UDP-Mpp instead of other nucleotide substrates utilized by the other UDP-d-*N*-acetylhexosamine: polyprenol phosphate d-*N*-acetylhexosamine 1-phosphate transferases [48].

Chung et al. used a chemical cross-linking experiment on detergent-solubilized AaMraY and structure-guided disulfide cross-bridge experiments (based on cysteines) on membrane-embedded AaMraY in order to show that AaMraY forms a dimer both in detergent micelles and in the membranes. It was suggested that this finding is in agreement with a previous study where bacterial two-hybrid studies were performed in the Gram-negative *Caulobacter crescentus* cells [49]. However, no clear indication in the original paper was found [49]. The fact that MraY was crystallized as a dimer and that dimerization of AaMraY was found in micelles and membranes was not given further explanation or analysis. Notably, the Gram-positive BsMraY comprises no cysteine residues. Remaining questions in this area include whether or not MraY always functions as a dimer, and how the oligomeric status influences the enzyme activity in different species would be an interesting topic for further studies on this system.

### 2.3. MraY Inhibitors and Inhibition Mechanism

MraY is regarded as an ideal target for novel antibiotics both because it is an essential molecular tool for generating the cellular envelope and because it has no counterparts in mammalian cells. A few different classes of MraY inhibitors have been studied to date. However, none of these has entered clinical development, to a large extent ascribed to the difficulties to deliver the compounds across the membranes. MraY inhibitors discovered thus far have come from either screening assays [50,51] or synthesis of compounds mimicking existing (natural) MraY inhibitors, such as mureidomycin A [52]. In this section, the methods for screening MraY inhibitors and the currently documented inhibitory compounds are discussed.

#### 2.3.1. Method Development for MraY Inhibitor Screening

Competitive binding to MraY was believed to be the key to the discovery of novel MraY inhibitors. The main assays designed to explore such compounds involved the use of a radiolabeled UDP-Mpp or a fluorescent variant of this substrate [27,51,53,54,55,56].

Formative experiments required the extraction of products or repeated filtration or washing steps that are not suitable for high throughput testing [57]. In other cases, the assays were coupled with other enzyme activities, such as MurG, transglycosylase or transpeptidase [54,55,58,59]. The first assay targeted only at MraY [57] is a microplate-based scintillation proximity assay (SPA) using a radiolabeled UDP-MurNAc-[^3^H]-propionate-pentapeptide. Unique features of this assay include: it used *E. coli* membranes instead of purified MraY, but still, the assay was selective towards MraY inhibition, as the radioactive product of the assay was identified as Lipid I, and the substrate can be synthesized easily in large quantity. A major disadvantage, however, is that 3 h of incubation of the wheat germ agglutinin (WGA)-coated SPA beads was necessary to capture the radioactive product, although the product was claimed to remain stable for up to 12 h.

The early assays used membrane preparations containing the lipid substrate C55-P implying that the activity of other enzymes, such as WecA, could not be excluded. Furthermore, radioactive waste disposal and equipment contamination are the general concerns when using a radioisotope-dependent assay. Later, a relatively inexpensive fluorescence-detection-based assay was developed that could avoid the involvement of any radioactive compounds [50]. In this assay, using the fluorescent substrate UDP-MurNAc-*N^ε^*-dansylpentapeptide (DNS-UDP-Mpp), the dansyl group is transferred to a hydrophobic environment upon synthesis of Lipid I, resulting in a blue shift and concomitant enhanced intensity of the fluorescent signal. This assay was validated by HPLC analysis where the product (DNS-Lipid I) could be clearly separated from the substrate (DNS-UDP-Mpp) [50]. Furthermore, a crude enzyme preparation of EcMraY-*Enterobacter cloacae* P99 β-lactamase protein fusion was used in this assay. The authors claimed that this protein fusion showed favorable activity over the wild-type EcMraY. This may raise the concern of whether the values determined in this assay represent the behavior of the MraY inhibitors correctly. IC_50_ values of known inhibitors such as mureidomycin B (IC_50_ = 0.038 µM) and synthetic riburamycin RU88110 (IC_50_ = 0.033 µM) determined by this assay were lower than those determined in an earlier cell-based assay that used toluene-permeabilized *E. coli* bacteria (0.065 µM and 0.33 µM, respectively) [60]. However, the IC_50_ value of tunicamycin determined in [50] (1.9 µM) was higher than by the cell-based assay (0.5 µM) [60]. The authors ascribed this to the difference in the mode of action of tunicamycin (reversible) with the other two compounds (slow binding) reported elsewhere [30] and/or its higher affinity to MraY in a membrane environment. In an early study, the MIC value of mureidomycin B was determined to be over 200 µg/mL (= 0.237 mM) against the *E. coli* NIHJ JC-2 strain, and its highest antibacterial activity was against the *Pseudomonas aeruginosa* SANK 70579 strain with an MIC value of about 0.24 µM [61]. These MIC values are not comparable with the IC_50_ values determined by either the fluorescence-based assay or the whole cell assay described above. This indicates the importance of cell/membrane penetration for inhibitors to reach their targets in order to be at their most effective [50,51,53,62].

**Figure 5:**
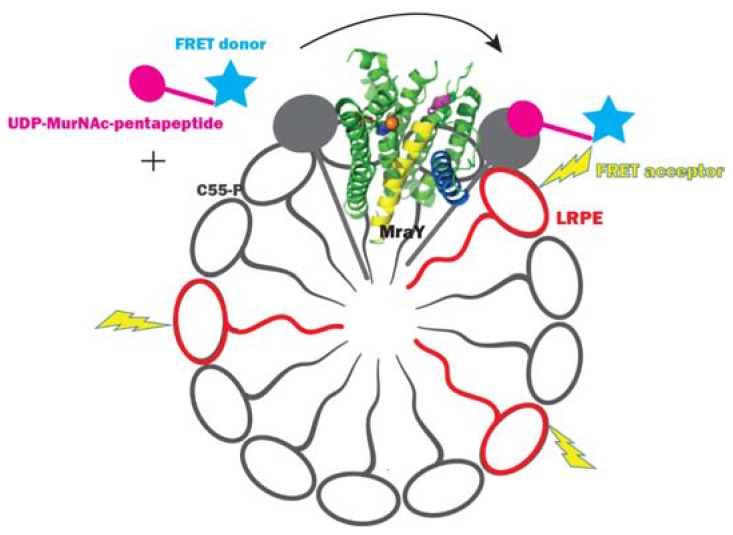
Fluorescence resonance energy transfer assay for MraY. The figure is adapted from [56].

Shapiro et al. [56] developed a fluorescence resonance energy transfer (FRET)-based assay to screen MraY inhibitors. In this assay, the FRET donor fluorophore BODIPY-FL was attached to labeled UDP-Mpp, and the FRET acceptor lipid lissamine rhodamine B dipalmitoyl phosphatidylethanolamine (LRPE) was embedded in the lipid micelle mix containing MraY and C55-P (Figure 5). When the UDP-Mpp labeled with the FRET donor is mixed with the detergent micelles containing active EcMraY and C55-P, the FRET donor is transferred to C55-P, yielding Lipid I labeled with the donor. Hence, the FRET donor and the acceptor are brought into close proximity yielding a FRET signal where the acceptor fluorescence increases when the donor fluorescence is excited. This assay used C55-P dissolved in detergent micelles and a membrane preparation of EcMraY. It was found that increase of C55-P concentration increased the sensitivity of the assay to inhibition by tunicamycin. This finding corresponds to the Km discrepancies of MraY between different studies when different C55-P concentrations were used, which is addressed as the major message in our own study [34]. This FRET assay allowed the measurement of the fluorescence intensity ratio of the donor and acceptor, hence fluctuations in measurements that influence both the donor and acceptor fluorescence could be ruled out. This way, the assay provided a more precise determination of percentage inhibition compared with the single fluorescence detection assay described above. This assay is sensitive to competitive inhibitors of UDP-Mpp. The IC_50_ values of tunicamycin determined by this assay were in the nanomolar range, apparently at odds with that determined by the other fluorescence assay described above. It should be noted that what proceeds in vitro, where both the substrate and the enzyme are dissolved in mixed micelles, is not comparable with what proceeds in vivo, where both C55-P and MraY are embedded in the membrane and can find each other through 2D diffusion. This means that discrepancies between IC_50_ and MIC values may be expected. The fact that MraY has a substrate embedded in the membrane, as well as a soluble nucleotide substrate presents a considerable challenge in designing a representative laboratory-based in vitro assay.

#### 2.3.2. Small Molecules that Inhibit MraY Activity

The best-known small molecule MraY inhibitors are the uridyl peptide antibiotics (UPAs), such as muraymycin, tunicamycin, mureidomycin, capuramycin and liposidomycin [27,30,31,41,63,64]. These small molecules share a common aminoribosyl-*O*-uridine skeleton, which was believed to be essential for their inhibitory activity, as it suggests that these compounds recognize and competitively bind to the UDP-Mpp binding sites on MraY [27,60,63,64,65]. A more detailed review has been published [27]. Here, we focus on the new discoveries, as well as a brief summary of the implications and challenges in developing such inhibitors.

Through detailed kinetics studies, UPA inhibitors were found to act against MraY in several ways. It was previously reported that mureidomycin A is competitive with both UDP-Mpp and C55-P, as the Km values of MraY for both substrates altered upon changing the inhibitor concentration [31]. This conclusion should be re-visited since the K_M_ value of either substrate is directly influenced by changing the concentration of the other [34]. This suggests that the inhibition of one substrate could indirectly influence the Km value for the other substrate without competitively inhibiting it. Tunicamycin was found to be only competitive with UDP-Mpp; liposidomycin B is non-competitive with UDP-Mpp [30]. It was also found that prolonged exposure of EcMraY to mureidomycin A [31] and liposidomycin B [30] did not alter the potency of these two inhibitors, indicating that the inhibition by these two molecules is reversible and that they are not consumed during the time of incubation, namely a slow-binding inhibitory mechanism.

Muraymycins (MRYs) are UPAs that have been studied extensively [65,66,67,68], and some exhibit antibacterial activity against Gram-positive pathogens, such as *S. aureus* and *Enterococcus* strains. MRY D2 and its epimer exhibit excellent anti-BsMraY activity with IC_50_ values of 0.01 µM and 0.09 µM, respectively, but both compounds showed very weak antibacterial activity against *S. aureus*, *E. faecalis* and *E. faecium* with MIC values over 64 µg/mL [65]. In contrast, two lipophilic analogues of MRY D2 with long lipid side chains showed excellent antibacterial activity against the same Gram-positive strains with MIC values between 0.5 and 4 µg/mL, although their IC_50_ values are about 10-fold higher than that of MRY D2 [65]. Apparently, adding lipophilic substituents [60,69,70,71] increased the accessibility of the active site of MraY for the MRY analogues, thereby improving their antibacterial activity. Further evidence remains to be established to explain this. A more recent study tested analogues of MRY D2 in the Gram-negative bacterium *P. aeruginosa*. Among all of the compounds tested, two analogues with a long lipid side chain and a positively-charged guanidinium group significantly increased the anti-*P. aeruginosa* activity [66]. Very recently, it was demonstrated through crystallography that AaMraY undergoes a large conformational change upon binding to MRY D2. This is the only structure of an MraY-inhibitor complex available at present. The structure shows that MraY is highly plastic and binds to its inhibitor MRY D2 in an overlapping, yet distinctive manner with respect to its natural nucleotide substrate. Such flexibility of MraY was also reported for MraY from *B. subtilis* [34], where it was demonstrated that BsMraY changes its conformation upon binding of C55-P to facilitate the nucleophilic attack on UDP-Mpp, involving an essential histidine residue.

How natural UPAs flip to the inner leaflet of cytoplasmic membrane and act against MraY as competitive inhibitors is not yet known. One hypothesis is that MraY is plastic and undergoes a conformational change upon periplasmic binding to UPAs. If correct, one could expect this to create a hydrophobic channel next to TM9, which may facilitate the uptake of these high molecular weight molecules to the cytoplasmic side and eventually allow them to bind to the active sites of MraY [63]. This hypothesis is based on some findings regarding the inhibition of EcMraY by protein E from bacteriophage ΦX174, which will be discussed in more detail in the next section. However, this implies that UPAs can bind to the periplasmic loops of MraY specifically, which seems not a likely mechanism.

Structural modification of known inhibitors provides a useful approach in developing novel inhibitors. An interesting example of this is the synthesis of 5′-triazole-substituted-aminoribosyl uridines (liposidomycin, caprazamycin or muraymycin) through Cu-catalyzed azide-alkyne cycloaddition (click chemistry) [72]. The 14 molecules investigated in this study exhibited IC_50_ values typically between 50 and 100 µM. Follow-up investigation using docking models showed that the activity of the most potent inhibitors correlates with their interaction with Leu191 in TM3 of AaMraY [73]. It was suggested that the introduction of a long lipid chain on the triazole substituent drastically improved the inhibitory activity. This lipid “anchor” may have helped in binding to TM3, given its possible involvement, hence locating the uridine close to the active sites of MraY. However, tests in vivo revealed that these molecules only showed bioactivity against Gram-positive bacteria with MICs between 8 and 32 µg/mL, including MRSA. Compared with the other studies on UPA-based MraY inhibitors, these two papers provide evidence for the involvement of an inhibition site (Leu191 of TM3) that was previously unknown.

Inhibitors developed through synthetic routes have only given high IC_50_ values thus far, e.g., 580 µM of an aminoribosyl-*O*-uridine based compound, limiting their application in vivo at present [72]. Nevertheless, a recently-discovered new MraY inhibitor through compound library screening, michellamine B, was found with a very high IC_50_ value (~0.5 mM against both EcMraY and BsMraY) and a reasonable MIC value (16 µg/mL against *B. subtilis*) [51]. Docking studies of the compound to the structure of AaMraY suggested that michellamine B inhibits MraY by binding to a hydrophobic groove formed between TM5 and TM9 of EcMraY (sequence alignment with MraY from other species shown in Figure 4). The authors addressed the importance of some phenylalanine residues in TM5 and TM9, which are known to make the TM interactions stronger [74]. It was also suggested that the presence of the polar glutamine residue that is close to a crucial phenylalanine (TM5) in *Staphylococcus aureus* MraY (SaMraY) was responsible for it becoming insensitive to michellamine B. However, this conclusion may require further evidence, as no mutant of these suggested residues was made to test this. Moreover, the docking study was carried out using the structure of AaMraY, while the antibacterial tests were performed on *B. subtilis*, which further complicates this issue. The possibility that MraY from different species may have different oligomeric states and that BsMraY is not necessarily present as a dimer have not yet been investigated. It is possible that the hydrophobic groove at the dimer interface of AaMraY proposed as the binding site of michellamine B may not even exist in BsMraY.

In summary, although some molecules show promising activity against MraY in tests carried out in vitro, applying these compounds in vivo, especially against Gram-negative pathogens, remains a challenge because keeping the compounds at low molecular weight while maintaining their antibacterial activity is not straightforward. Furthermore, some molecules, e.g., tunicamycin, possess other disadvantages, such as inhibiting other targets, e.g., GlcNAc-1-phosphate transferases, that are present in mammalian cells, which have hampered their clinical development [30,75,76].

#### 2.3.3. MraY Inhibition by ΦX174 Protein E

Bacteriophages provide another therapeutic source of antibiotic lead compounds to treat bacterial infections. Early studies have found that protein E of a small single-stranded DNA phage ΦX174 causes *E. coli* cell lysis [77]. The exact mechanism was later revealed that protein E interferes with MraY function in *E. coli* and disrupts peptidoglycan synthesis [78]. Interestingly, this E-mediated lysis does not occur in Gram-positive bacteria [79].

**Figure 6:**
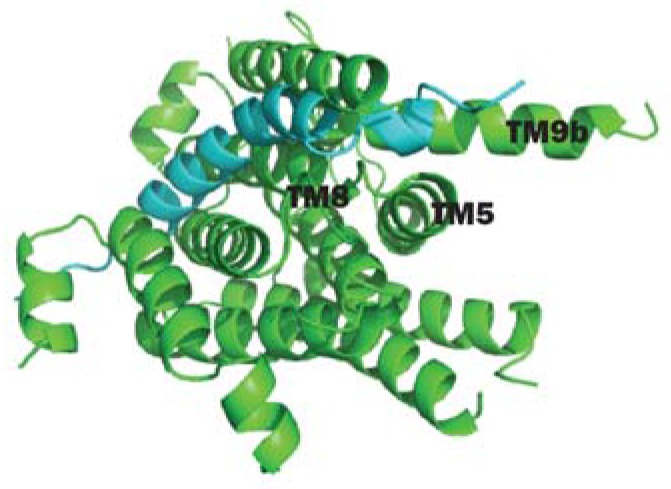
Cytoplasmic view of the docking model of protein E (the light blue helix) to an *E. coli* MraY structure model (modeled based on 4J72.pdb). The protein E helix binds closely to the hydrophobic groove formed by TM5, TM8 and TM9 and is bent towards the TM9b. The figure is rendered with Pymol.

Protein E is a 91-amino acid membrane protein with one transmembrane helix with 35 amino acids followed by a cytoplasmic domain. Both genetic and biochemical studies have found that the 29 amino acids from its N-terminus, which form the transmembrane domain of protein E, are responsible for the inhibition of MraY [77,80,81,82,83]. E is non-competitive with either substrate of MraY. Site-directed mutagenesis revealed that a proline at the 21 position of the TM domain of E is critical for its lytic activity; moving this proline along the membrane helix resulted in it being unable to inhibit MraY, suggesting that the kink at position 21 in the protein E helix caused by the proline is absolutely crucial [82]. It is unclear which binding pocket or helix of MraY binds to protein E because a structural model of MraY bound to protein E has not yet been produced. Genetic screening showed that Phe288 in TM9 of EcMraY (Phe286 on TM9 in AaMraY, Figure 5) is essential for its sensitivity towards protein E, as a single site mutation of F288L caused resistance against protein E. However, this phenylalanine residue is however missing in the E-insensitive Gram-positive BsMraY and SaMraY (Figure 4). TM9 is a titled helix in the AaMraY structure that breaks into two helices (TM9a and TM9b), while E has a kink caused by a proline [8,82]. In this respect, it is interesting to note that docking studies of protein E with a structural model of EcMraY suggested that TM9 (near the dimer interface) of MraY is involved in the binding to protein E indicating that this kinking of the helices may be important for their mutual interaction (Figure 6 [84]). In terms of a hypothesis, MraY presents a unique configuration when bound to protein E. In addition, we observed that there was no favored binding of E to the dimer interface when we tried to dock protein E to a structural model of BsMraY. This is an interesting observation given that BsMraY is not inhibited by protein E. Again, it is not clear whether BsMraY functions as a dimer at all in vivo. The reason why protein E does not interact strongly with the Gram-positive MraY homologues remains unresolved. Rodolis et al. [64] synthesized small peptides sharing a partial sequence of protein E and explored their inhibition of MraY homologues from both Gram-positive and -negative species. It was reported that an RWXXW motif found in E and other cationic antimicrobial peptides is essential for MraY inhibition. However, the small synthetic peptides reported in this paper all have a much lower inhibition, with an MIC value about 10–20-fold higher than protein E or the synthetic Epep (with the first 37 amino acid residues of protein E) against *E. coli*. Another study from the same group [63] presented the hypothesis that some UPAs, which show structural resemblance to the RWXXW motif, may bind to the protein E binding site on MraY near the periplasmic side initially before crossing the membranes and eventually bind to the active sites of MraY. Yet, evidence for this is lacking. Besides, protein E is synthesized in the cytoplasm of bacterial cells, while the other small molecule inhibitors of MraY must penetrate the cell envelope. This may result in a completely different mode of action in terms of how and where the molecules start binding to MraY. Therefore, it may be simplistic to attribute the inhibitory activity of protein E to a short peptide sequence.

MraY remains an interesting target for antibiotic discovery and development, although it still faces many challenges, particularly the penetration of the cell envelope. This holds true for all of the antibiotic targets that reside in the bacterial cytosol. The Lipid I synthesis catalyzed by MraY in vivo is drawn by the subsequent reaction of Lipid II synthesis, for which MurG is the responsible enzyme. Interestingly, these two enzymes interact as was shown by co-immunoprecipitation experiments [85]. In the following section, we will review its discovery, characterization and the advances made in inhibiting this important bacterial enzyme.

## 3. MurG

The earliest work on the *murG* gene indicated that it was involved in the cell envelope biogenesis and, in particular, peptidoglycan metabolism [86]. Later, it was identified that *murG* codes for an *N*-acetylglucosamine (GlcNAc) transferase, from then on referred to as MurG [87]. This enzyme belongs to the glycosyltransferase family and catalyzes an irreversible essential step on the membrane after MraY. MurG attaches the GlcNAc from UDP-GlcNAc to Lipid I and produces Lipid II [88]. MurG is an essential enzyme and is conserved across almost all bacterial species, which makes MurG a great target for novel antibiotics. However, MurG is a paradigm for glycosyltransferases that are present in the vast majority of both prokaryotic and eukaryotic cells. This implies that only inhibitors that compete with Lipid I bear the potential of further clinical development. The structural information regarding the substrate selectivity and the inhibition mechanism of MurG will be discussed in the following sections.

### 3.1. Biochemical Characterization of MurG

An early study demonstrated that *E. coli* MurG (EcMurG) is a membrane-associated protein [87]. Later, it was reported that EcMurG is exposed to the cytoplasm of *E. coli* by showing that the enzyme in spheroplasts was not sensitive to trypsin treatment [89]. With this knowledge combined, it could be concluded that MurG is peripherally associated with the inner leaflet of the plasma membrane.

Crouvoisier et al. described a purification of EcMurG to greater than an 80% yield using immobilized affinity beads [90], suggesting that the production and purification of MurG was much easier compared with the membrane-embedded MraY. N-terminally His-tagged MurG showed higher yield and purity with respect to wild-type or C-terminally His-tagged MurG. The authors concluded that MurG is a peripheral membrane protein according to a few criteria: partial solubilization by salt treatments, purification without detergent, localization on the inner side of the cytoplasmic membrane, a cationic theoretical pI value of 9.7 and a lack of significant hydrophobic regions in its amino acid sequence. It was yet unknown how exactly MurG associates with the bacterial membrane.

**Figure 7:**
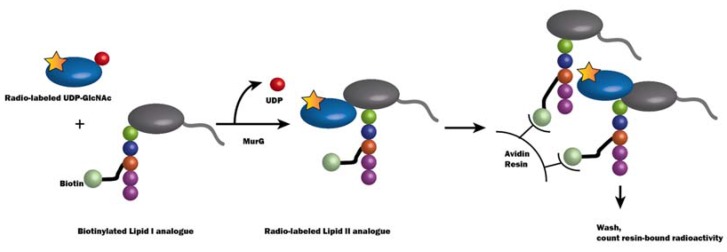
Schematic illustration of the biotin-capture assay for MurG activity [91]. In this assay, MurG catalyzes a reaction between radiolabeled (depicted by a yellow star) UDP-GlcNAc and biotinylated Lipid I analogue, and Lipid II with the radiolabel is synthesized. The avidin resin, which binds to biotin, therefore catches the radioactivity, which can be counted to indicate the MurG activity.

The kinetics of MurG had been difficult to measure because the lipid substrate Lipid I typically has a low abundance in vivo [54]. A series of water-soluble Lipid I analogues were used to determine EcMurG activity and kinetics in a biotin-capture assay (Figure 7) [91]. In this assay, a radiolabeled UDP-(^14^C)-GlcNAc and a functional biotinylated GlcNAc acceptor analogue of Lipid I were incubated together with cell lysate enriched in MurG enzyme for a period of time. The reaction was quenched by adding SDS to a final concentration of 0.33%. Radioactivity was captured by an avidin-derivatized resin and counted after the unbound radioactivity was washed away.

**Figure 8:**
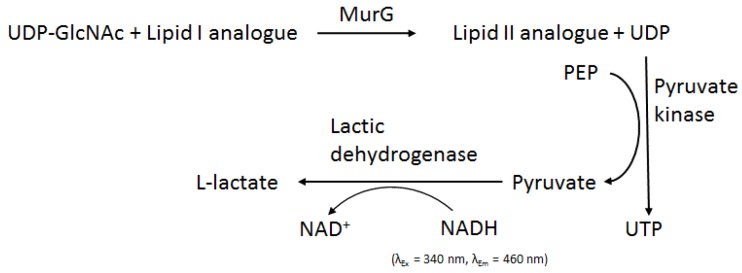
Fluorescence coupled assay for MurG. The figure is adapted from [88].

Production of pure MurG without the need of using detergent [90] led to speculation that MurG activity did not require membranes and could be assayed with soluble substrates instead of the natural long chain Lipid I [88]. A fluorescence assay for MurG coupled with pyruvate kinase activity was developed [88], which measures the decrease of the fluorescence signal from NADH (Figure 8). This study revealed that MurG accepted soluble substrates with short chains (two prenyl units) and preferred substrates that have a *cis*-allylic double bond. These findings built the foundation of using synthetic soluble substrates (Lipid I mimics) to assay MurG activity. It should be noted that in such a reaction system, MurG is not associated with any membranes, unlike its natural status. This will require that the Lipid I substrate finds the enzyme via three-dimensional diffusion. In vivo, Lipid I, and MurG likely as well, is membrane bound. Hence, they are able to find each other via two-dimensional diffusion, which is much more effective than diffusion in three dimensions, which normally occurs in solutions [92].

Later, it was reported that lipid vesicles enriched in the negatively-charged cardiolipin co-purified with EcMurG and the presence of cardiolipin also enhanced MurG activity [93]. This study provided the first direct evidence that MurG was linked to the cytoplasmic membrane by direct interaction with lipids and preferably with cardiolipin.

Besides the (naturally-occurring) polyisoprenyl-bound Lipid I mentioned above, it was also reported that the saturated C14 alkyl-Lipid I was a substrate of MurG, which was even the best performing analogue in this study [94]. Nevertheless, both studies showed that MurG prefers shorter Lipid I analogues than its natural Lipid I substrate in an in vitro set-up, possibly due to solubility problems because of the long lipid chains and the 3D diffusion mode in vitro instead of the 2D diffusion mode in vivo, as mentioned above. This is the first assay for MurG that did not rely on end point measurement or any radioisotope.

### 3.2. Structural Characterization of MurG

Soon after the first report on its purification, the crystal structure of EcMurG at 1.9 Å (PDB ID: 1F0K) emerged [95]. The crystal structure revealed two major domains of MurG and a hydrophobic cleft formed in between the domains. Although the homology is relatively low, the two domains of MurG are structurally similar. Both domains have Rossman-like folds, which is typical for nucleotide binding domains [96,97]. In particular, the C-domain of EcMurG shares significant structural homology with the C-domain of phage T4 β-glucosyltransferase (BGT) containing a UDP binding pocket. It is therefore suggested that the C-domain of MurG is the binding site of UDP-GlcNAc and transfers the GlcNAc moiety to Lipid I. Moreover, it was proposed that MurG is associated with the negatively-charged bacterial membrane via a hydrophobic patch surrounded by basic residues through hydrophobic and electrostatic interactions, which inspired the later finding that MurG associates with the membrane preferably via cardiolipin, described in Section 3.1 [93].

A co-crystal structure of EcMurG with UDP-GlcNAc (PDB ID: 1NLM) [98] confirmed that UDP-GlcNAc binds tightly to the C-domain. Moreover, substrate specificity studies revealed that EcMurG is highly selective for the nucleotide attached to its donor sugar substrate unlike most other GTases that do not discriminate between UDP and TDP [99]. EcMurG showed no activity when TDP-GlcNAc was used as the substrate [98]. The affinity of MurG to other nucleotide diphosphates, such as CDP, ADP and GDP, is at least 10-times lower than to UDP [91]. This substrate specificity may be sufficient for developing inhibitors for the UDP-binding site on MurG.

Many conserved residues of MurG are located in between the two major domains situated in the cleft. Crouvoisier et al. [100] aligned over 70 MurG orthologues from different bacterial species and performed site-directed mutagenesis to explore the functional significance of those conserved residues. Their studies have identified 13 residues located near the cleft that are somehow important for MurG’s activity. The mutation of these amino acids into alanine has either caused significant loss of MurG activity or resulted in a highly unstable protein.

**Figure 9:**
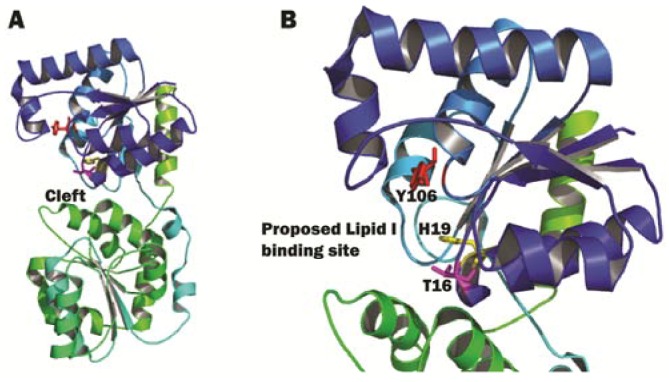
*E. coli* MurG structure. (**A**) The complete view of *E. coli* MurG, N-domain in blue, C-domain in green. The cleft in between the two domains is indicated by an arrow; (**B**) A close-up view of the cleft between the N- and C-domains of MurG. Residues T16 (pink), H19 (yellow) and Y106 (red) are shown as sticks. The proposed Lipid I binding site is indicated by an arrow. The image was obtained and rendered using Pymol.

The different MurG homologues have moderate sequential homology, while their structural homology is thought to be high among different species that make peptidoglycan. This was demonstrated when the structure of *P. aeruginosa* MurG (PaMurG) bound to UDP-GlcNAc was also solved by a different group [101]. Although the sequence homology between these two MurG homologues was only 45%, the structures and the mode of binding to the donor substrate are very similar. The authors noted that one major difference between the structures was that the N-domain of *E. coli* MurG swung further away from the C-domain, causing the cleft between the two domains to be larger than that of PaMurG. For EcMurG, UDP-GlcNAc is situated closely to the cleft, which is not large enough to accommodate TDP-GlcNAc. An even narrower cleft in PaMurG confirms the substrate specificity regarding the nucleotide, but this knowledge does not seem to add any other significance, since EcMurG already has a sufficiently narrow cleft that facilitates only specific binding to UDP other than other nucleotide or nucleoside substrates. In the structure of PaMurG, H15 (equivalent of H19 in EcMurG) is present, as well. This residue is not only positioned very close to the bound UDP-GlcNAc, but also points towards the proposed Lipid I binding site (Figure 9B). This indicates the importance of this histidine residue in coupling the donor sugar to the acceptor Lipid I. To date, there is no crystal structure of MurG (from any bacterial species) bound to Lipid I available. Research in understanding the precise nature of the Lipid I-substrate interaction is essential for furthering our understanding of the mode of action of this enzyme.

### 3.3. MurG Inhibitors

To date, the discovery of selective MurG inhibitors typically relies on two approaches. One approach is to synthesize UDP-GlcNAc-mimicking compounds either by elaborating the nucleotide group or to use existing inhibitors to design similar molecules. The alternative approach relies on screening of a compound library to generate leads that can competitively bind to MurG using an assay that exploits the purified enzyme [98,102,103]. The compounds discovered through this channel require further modification to avoid two scenarios: (1) binding to other glycosyltransferases that are also present in eukaryotic cells; (2) poor penetration of the cell wall.

Since the elucidation of the structure of MurG-UDP-GlcNAc, efforts to identify competitive inhibitors by screening analogues of UDP-GlcNAc-mimicking compounds have been under way [103]. The ligand-bound MurG structure revealed that the methyl of the *N*-acyl group of UDP-GlcNAc was exposed, which suggests that modification of this group will not affect the binding of UDP-GlcNAc to MurG. A fluorescently-labeled UDP-GlcNAc was made based on this hypothesis. It was found that the fluorescent modification slowed rather than abolished substrate binding. The fluorescence decreased when the binding was inhibited by the addition of MurG inhibitors, as the substrate was displaced from MurG. A high throughput screening based on the substrate displacement was established. Using this method, the authors screened 64,000 molecules that came from a variety of compound libraries, and less than 0.6% of the molecules were found as hits. To validate the selectivity, kinetics assays as described in Section 3.1 (Figure 7) were used, and eventually, seven compounds were identified as selective MurG inhibitors with IC_50_ values ranging between 1 and 7 µM. However, all seven compounds showed an inhibitory effect against another glycosyltransferase GtfB (structurally related to MurG), with IC_50_ values of between two- and 10-fold higher. This indicates that the UDP-binding site is a relatively poor target for inhibitors despite MurG’s specificity for UDP-GlcNAc over TDP-GlcNAc (vide supra). This suggests that the substrate displacement assay can only be used for identifying hit compounds. Inhibitor selectivity must be verified using another assay that is specific for MurG. However, such specificity will have to rely on blocking the binding of Lipid I to MurG.

Another interesting assay is based on a dansylated Lipid I analogue (MurNAc(*N*ε-dansylpentapeptide)-pyrophosphoryl (*R*, *S*)-α-dihydroheptaprenol, C35-Lipid I) and a radiolabeled UDP-(^14^C)-GlcNAc [104]. The radioactive substrate and product were separated using reverse-phase HPLC. Although this assay was shown to be rapid and specific for MurG, the authors could not separate the product (Lipid II) from the substrate (Lipid I) based on the dansyl fluorescent label. This assay could therefore not be optimized to a high throughput format easily. Nevertheless, this study showed for the first time that, via observations of the transfer of the radiolabel, a reverse reaction from Lipid II and UDP to UDP-GlcNAc can happen at a very low rate.

Several MraY-MurG coupled assays were also made available to discover lead compounds that can inhibit either or both enzymes in vitro [54,55,58,59]. Besides a higher efficiency in finding useful leads, the other advantage of such an assay is that there is no need to synthesize Lipid I analogues prior to the assay and thereby lower the cost.

It was reported that a vancomycin derivative with *N-*chlorobiphenyl-*N-*methyl leucine was a potent inhibitor of MurG in vitro [94]. Moenomycin, a known antibiotic that interferes with the function of the transglycosylase domain of PBP1B [105], was also found to inhibit MurG. However, neither vancomycin nor moenomycin inhibits MurG in vivo, as neither can penetrate the bacterial membranes and, therefore, do not encounter MurG [94].

The most recently-found inhibitor of MurG is a narrow-spectrum compound called murgocil [106]. While screening for antibacterial compounds that inactivate MRSA, the steroid-like molecule murgocil was identified to bind to MurG specifically. An assay in vitro showed that Lipid II synthesis was inhibited by murgocil in a dose-dependent manner. Interestingly, the bioactivity of murgocil against *Staphylococci* (MIC = 2–4 µg/mL) was considerably higher than its activity in vitro against purified *S. aureus* MurG (IC50 = 115 µM ≈ 51 µg/mL). Taken together with docking studies of the homology model of SaMurG based on the EcMurG structure, murgocil appears to inhibit peptidoglycan synthesis more efficiently in whole cells and has a synergistic activity with a β-lactam partially by delocalizing PBP2 from the division septum during peptidoglycan synthesis. The binding site of murgocil to MurG was revealed by several murgocil-resistant staphylococci of which the resistance could be mapped in the previously-mentioned cleft region between the N- and C-domains of MurG. Docking studies confirmed that murgocil can bind to this cleft of MurG and may lock the enzyme in an inflexible conformation. The significantly higher IC_50_ value of murgocil may be ascribed to the difference between the measurements taken from in vitro and in vivo systems. Lipid I is always embedded in the phospholipid bilayer membranes in vivo, where MurG can reach it via lateral diffusion. This is not the case for activity tests in vitro, where the substrate Lipid I is often presented in micelles, and in some cases, water-soluble Lipid I analogues are used [107]. Again, murgocil activity in vivo is strictly restricted to *Staphylococci*, the possibility of expanding its use to other Gram-positive or -negative bacteria seems rather limited, since it was found that murgocil activity depends on some amino acid residues that are unique in SaMurG, including M45 and D168. However, further work is required to confirm this.

The development on MurG inhibitors has reached a plateau by comparison to MraY. The potential toxicity of MurG inhibitors in vivo that compete with UDP-GlcNAc remains the biggest concern in terms of further clinical development.

## 4. Conclusions

Recent work has led to a substantial growth in our understanding of the structural and biochemical characteristics of MraY and MurG, which led to the gradual unraveling of the mode of action of these enzymes. It is evident that previous studies have put much effort in investigating the nucleotide substrate of both MraY and MurG, while many questions regarding the lipid substrate for both enzymes have yet to be answered. Once we understand the molecular details of their mutual interaction, novel inhibitors can be designed to block the enzymatic activity. The search for combination therapies that involve a synergistic effect of a cell wall inhibitor with another class of antibiotics, e.g., a β-lactam, might form part of the therapy yet to come.

## Supplementary Materials

Supplementary File 1
