# Supplementary material for: The Membrane Steps of Bacterial Cell Wall Synthesis as Antibiotic Targets
Source: Antibiotics (Basel). 2016 Aug 26;5(3):28. doi: 10.3390/antibiotics5030028 (PMC5039524; doi:10.3390/antibiotics5030028)
Supplement: Supplementary File 1 [file antibiotics-05-00028-s001.pdf]

# Supplementary Materials: The Membrane Steps of Bacterial Cell Wall Synthesis as Antibiotic Targets

**Yao Liu and Eefjan Breukink**

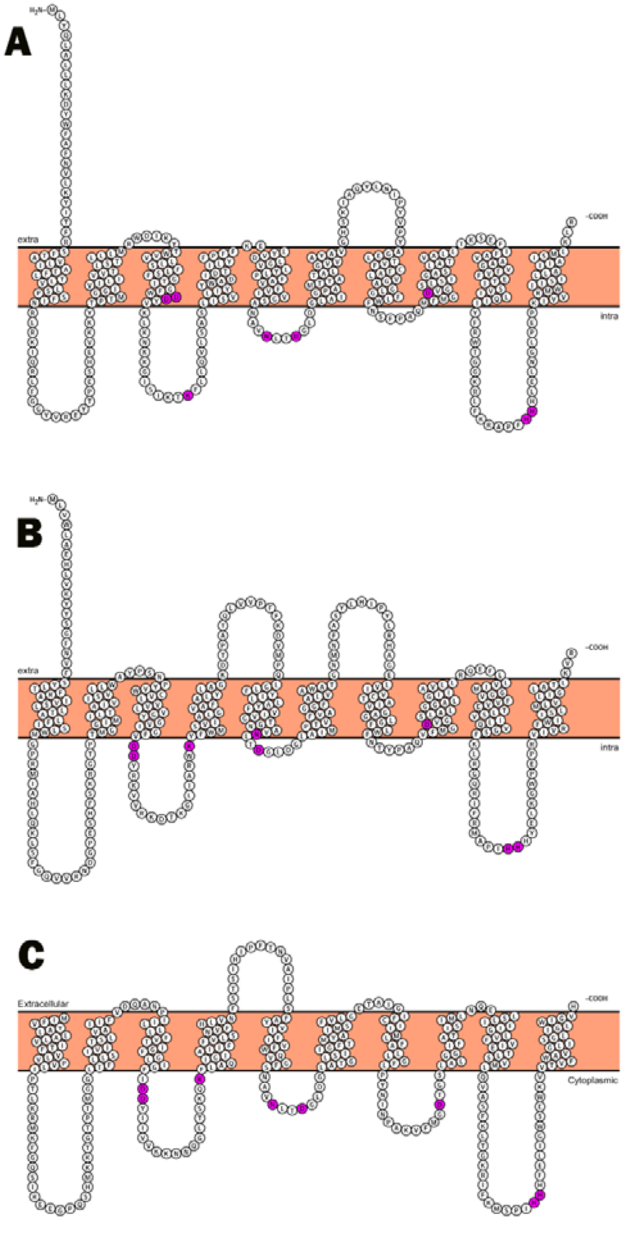


**Figure S1.** Topology maps of (**A**) *A. aeolicus* MraY, (**B**) *E. coli* MraY, and (**C**) *S. aureus* MraY. Highly conserved residues (equivalents of D98, D99, K116, N171, D174, D231, H289 and H290 of *B. subtilis* MraY) are highlighted in pink. The figures are rendered with Protter webservice.

© 2016 by the author; licensee MDPI, Basel, Switzerland. This article is an open access article distributed under the terms and conditions of the Creative Commons Attribution (CC-BY) license (http://creativecommons.org/licenses/by/4.0/).
